# Quality of T-Cell Response to SARS-CoV-2 mRNA Vaccine in ART-Treated PLWH

**DOI:** 10.3390/ijms232314988

**Published:** 2022-11-30

**Authors:** Eeva Tortellini, Maria Antonella Zingaropoli, Giulia Mancarella, Raffaella Marocco, Anna Carraro, Meriem Jamhour, Christian Barbato, Mariasilvia Guardiani, Federica Dominelli, Patrizia Pasculli, Anna Napoli, Aurelia Gaeta, Fabio Mengoni, Paola Zuccalà, Valeria Belvisi, Blerta Kertusha, Alberico Parente, Cosmo Del Borgo, Vincenzo Vullo, Maria Rosa Ciardi, Claudio Maria Mastroianni, Miriam Lichtner, LATINA COVID-19 Group

**Affiliations:** 1 Department of Public Health and Infectious Diseases, Sapienza University of Rome, 00185 Rome, Italy; 2 Infectious Diseases Unit, SM Goretti Hospital, Sapienza University of Rome, 00185 Latina, Italy; 3 Institute of Biochemistry and Cell Biology (IBBC), National Research Council (CNR), Department of Sense Organs, Sapienza University of Rome, 00185 Roma, Italy; 4 Department of Molecular Medicine, Sapienza University of Rome, 00185 Rome, Italy; 5 Department of Neurosciences, Mental Health, and Sense Organs, NESMOS, Sapienza University of Rome, 00185 Rome, Italy

**Keywords:** SARS-CoV-2, T-cell response, humoral response, PLWH, mRNA vaccine, flow-cytometry, HIV

## Abstract

We investigated specific humoral and T-cell responses in people living with HIV (PLWH) before (T0), after two (T1) and after six months (T2) from the third dose of the BNT162b2 vaccine. Healthy donors (HD) were enrolled. The specific humoral response was present in most PLWH already after the second dose, but the third dose increased both the rate of response and its magnitude. Collectively, no significant differences were found in the percentage of responding T-cells between PLWH and HD. At T0, stratifying PLWH according to CD4 cell count, a lower percentage of responding T-cells in <200 cells/µL subgroup compared to >200 cells/µL one was observed. At T1, this parameter was comparable between the two subgroups, and the same result was found at T2. However, the pattern of co-expression of IFNγ, IL2 and TNFα in PLWH was characterized by a higher expression of TNFα, independently of CD4 cell count, indicating a persistent immunological signature despite successful ART. mRNA vaccination elicited a specific response in most PLWH, although the cellular one seems qualitatively inferior compared to HD. Therefore, an understanding of the T-cell quality dynamic is needed to determine the best vaccination strategy and, in general, the capability of immune response in ART-treated PLWH.

## 1. Introduction

Since December 2019, the Severe Acute Respiratory Syndrome CoronaVirus-2 (SARS-CoV-2) has spread globally, causing a devastating pandemic that has posed enormous healthcare challenges around the world [1]. Many countries, Italy included, have launched vaccine campaigns against coronavirus disease 2019 (COVID-19) [2]. Among the vaccines based on different technologies that have been developed in the last period, the mRNA vaccine mRNABNT162b2 (Comirnaty^®^) has been widely used in Italy, in addition to the immunization of frail and immunosuppressed individuals [2].

A growing number of studies demonstrate that mRNA-based vaccines induce a robust and protective humoral and cellular response against SARS-CoV-2 Spike protein (S), highlighting how vaccination has proven to be an effective method to reduce mortality and morbidity related to SARS-CoV-2 infection [3,4,5,6]. In particular, there is increasing evidence of the importance of vaccine-induced S-specific T-cells in conferring protection against COVID-19, as observed for those induced by natural infection in convalescent individuals [7,8,9,10]. In fact, vaccine-induced S-specific T-cells are capable of recognizing different regions of S protein, contributing to vaccine efficacy against viral variants and explaining why they often cause only mild or asymptomatic disease in individuals who have completed a two-dose vaccination regimen [8,9,10].

Most of the data available regarding immunocompetent individuals and vaccination of immunosuppressed individuals remain challenging, evidencing the need to further investigate their immune response against vaccines in general, including the mRNA-based COVID-19 vaccine.

In fact, people living with human immunodeficiency virus (PLWH) may incur a higher risk of developing severe COVID-19 [11]. In particular, cellular immune deficiency and a lower CD4 cell count/low CD4 T-cell nadir have been identified as potential risk factors for severe SARS-CoV-2 in this population, in accordance with the evidence of the important role of CD4 in resolving SARS-CoV-2 infection [11,12,13].

Although the effective control of viremia with antiretroviral therapy (ART) is correlated with an improvement of responsiveness to routine vaccines, some immune defects like inflammation and immune activation that determine the comorbid conditions associated with premature aging in PLWH might alter the vaccine-induced immune response to SARS-CoV-2 [14,15,16].

In particular, the studies available that focus on the response to mRNA-based COVID-19 vaccines among PLWH show values of anti-S antibodies, neutralizing antibody activity and cellular immune responses comparable to those observed in healthy donors [13,17,18,19,20]. More recently, a lower current CD4 cell count has been associated with inferior responsiveness to vaccination in this population [21].

However, reports regarding the immune response in PLWH are not always in accordance, and information about the quality of T-cell response in this population is still lacking. Therefore, the aim of the present study was to evaluate specific humoral and T-cell responses before and after the administration of the third dose of mRNA vaccine Comirnaty^®^ in a cohort of PLWH, according to the current CD4 cell count, with a focus on the quality of T-cell response.

## 2. Results

### 2.1. Demographic and Clinical Characteristics of PLWH

Overall, 37 PLWH and 18 HD were enrolled in the study. Nadir and current median CD4 cell count (IQR) of PLWH were 90 (22–281) cells/µL and 547 (308–714) cells/µL, respectively. Median HIV-1 viral load (IQR) was 40 (40–166) cp/mL (Table 1).

As reported in Table 1, all PLWH were on ART at the time of SARS-CoV-2 vaccination. Specifically, 22% were on a dual ART regimen, and 78% were on a non-dual ART regimen. Concerning comorbidities, all PLWH had at least one coexisting illness, and among them, 70% had more than one. Regarding HCV serostatus, 24% of PLWH were anti-HCV Ab positive. Finally, 65% of enrolled PLWH were not smokers (Table 1).

Specifically, 31 PLWH and 12 HD were enrolled before the third dose of mRNA vaccine administration, while 28 PLWH and 15 HD were enrolled after the third dose. Finally, a supplementary time point for 27 PLWH was included (T2) (Table S1).

### 2.2. Evaluation of Spike-Specific Humoral and T-Cell Response in Study Population

The evaluation of S-specific humoral response was performed in 37 PLWH and 12 HD at T0 and 31 PLWH and 15 HD at T1.

Overall, 86% (32/37) and 94% (29/31) of PLWH showed detectable levels of anti-S antibodies at T0 and T1 versus 100% of HD at both time points.

**Figure 1:**
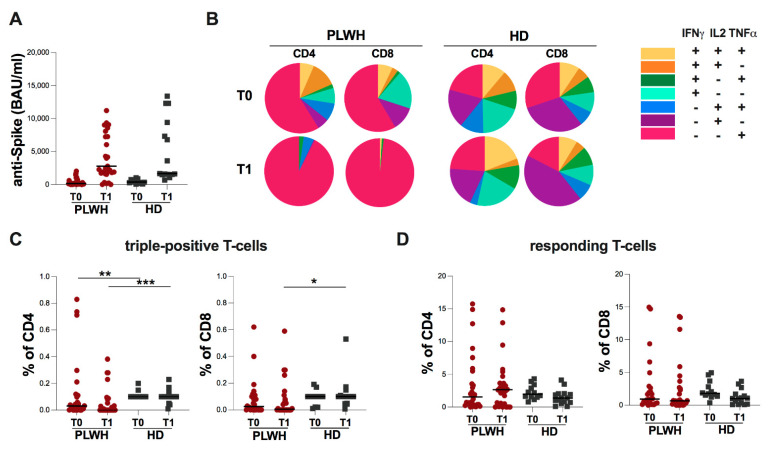
Evaluation of S-specific humoral and T-cell response in PLWH and HD at T0 and T1. (**A**) Anti-S antibody levels in PLWH and HD. For each time point, the differences between the two groups were evaluated using the nonparametric Mann-Whitney test. Data are shown as median (lines). (**B**) Pie charts representing multifunctional cytokine analysis of specific T-cells in PLWH and HD. (**C**) Percentage of triple-positive T-cells in PLWH and HD. The differences were evaluated using the nonparametric Mann-Whitney test. Data are shown as median (lines). (**D**) Percentage of responding T-cells in PLWH and HD. The differences were evaluated using the nonparametric Mann-Whitney test. Data are shown as median (lines). Statistical significance *(p*) is reported in the graphics. *** *p* < 0.001; ** *p* < 0.01; * *p* < 0.05.

The cross-sectional evaluation showed no significant differences in anti-S antibody levels between the two groups at both time points (Figure 1A).

Furthermore, the longitudinal evaluation of 31 PLWH and nine HD showed a significant increase in anti-S antibody levels at T1 compared to T0 in both groups (PLWH: *p* < 0.0001; HD *p* = 0.0039).

Regarding the T-cell specific response, an uneven T-cell subset distribution in PLWH at both time points was observed, with a predominance of monofunctional T-cells producing TNFα (IFNγ-IL2-TNFα+), while in HD, a heterogeneous distribution of T-cell cytokines producers was found (Figure 1B).

Indeed, when comparing T-cell CD4 subset distribution at both T0 and T1 in PLWH and HD, a statistically significant difference was observed (*p* = 0.0562 and *p* = 0.0018, respectively) as well as in T-cell CD8 subset distribution at T1 (*p* = 0.0001) (Figure 1B).

Specifically, a lower percentage of CD4 triple-positive T-cells was found in PLWH compared to HD at both time points (T0: *p* = 0.0076; T1: *p* = 0.0003) (Figure 1C) as well as a lower percentage of CD8 triple-positive T-cells at T1 (*p* = 0.0127) (Figure 1C).

Finally, at both time points, no statistically significant differences in the percentage of responding T-cells in PLWH compared to HD were found (Figure 1D).

All medians and [IQR] are reported in Table S2.

### 2.3. Evaluation of Spike-Specific Humoral and T-Cell Response in PLWH Stratified According to CD4 Cell Count

**Figure 2:**
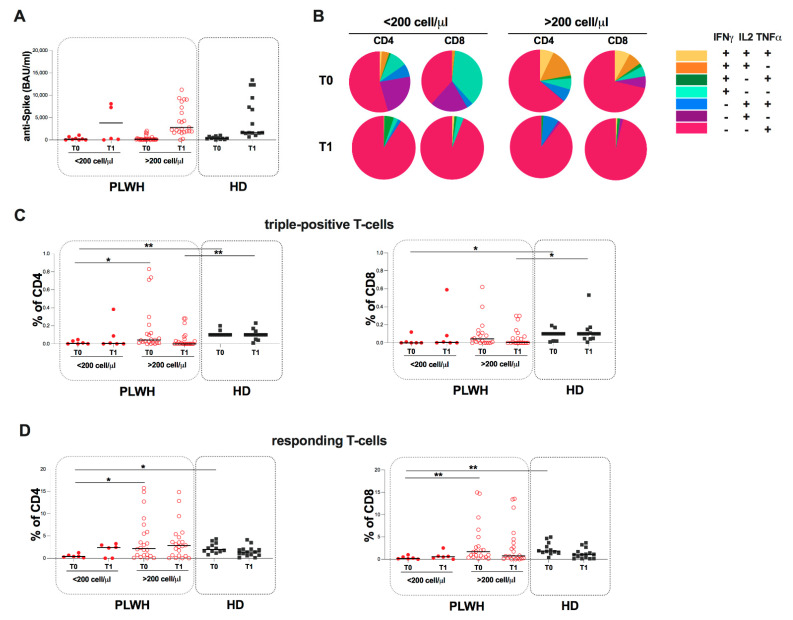
Evaluation of S-specific humoral and T-cell response in PLWH stratified according to CD4 cell count at T0 and T1. (**A**) Anti-S levels in PLWH stratified according to CD4 cell count. The differences between PLWH with <200 and PLWH with >200 subgroups were evaluated using the nonparametric Mann-Whitney test. Both CD4 > 200 and CD4 < 200 groups were compared to HD using the nonparametric Kruskal-Wallis test with Dunn’s post-test. Data are shown as median (lines). (**B**) Pie charts representing multifunctional cytokine analysis of specific CD4 and CD8 T-cells in PLWH stratified according to CD4 cell count. (**C**) Percentage of triple-positive T-cells in PLWH stratified according to CD4 cell count. The differences between CD4 > 200 and CD4 < 200 groups were evaluated using the nonparametric Mann-Whitney test. Both CD4 > 200 and CD4 < 200 groups were compared to HD using the nonparametric Kruskal-Wallis test with Dunn’s post-test. Data are shown as median (lines). (**D**) Percentage of responding T-cells in PLWH stratified according to CD4 cell count. The differences between CD4 > 200 and CD4 < 200 groups were evaluated using the nonparametric Mann-Whitney test. Both CD4 > 200 and CD4 < 200 groups were compared to HD using the nonparametric Kruskal-Wallis test with Dunn’s post-test. Data are shown as median (lines). Statistical significance (p) is reported in the graphics. ** *p* < 0.01; * *p* < 0.05.

According to CD4 cell count, PLWH were stratified into two subgroups: PLWH with <200 cells/µL (six males and two females, median age [IQR] 47 [43.5–55]) and PLWH with >200 cells/µL (20 males and nine females, median age [IQR] 63 [57–68.5]). At both time points, no differences were found in anti-S antibody levels between the two subgroups, as well as comparing the two subgroups to HD (Figure 2A).

Regarding the T-cell specific response, an uneven T-cell subset distribution in <200 cells/µL and >200 cells/µL at both time points was observed. In particular, looking at the pie charts, the proportion of monofunctional T-cells producing TNFα (IFNγ-IL2-TNFα+) are the most represented with respect to the other subpopulations (Figure 2B).

Specifically, at T0, comparing <200 cells/µL and >200 cells/µL subgroups, a lower percentage of CD4 triple-positive T-cells in PLWH with <200 cells/µL was observed (*p* = 0.0435) (Figure 2C).

Comparing the two subgroups to HD, a lower percentage of triple-positive T-cells was found in PLWH with <200 cells/µL compared to HD (CD4: *p* = 0.0017; CD8: *p* = 0.0312) (Figure 2C).

At T1, a lower percentage was found in >200 cells/µL compared to HD (CD4: *p* = 0.0012; CD8: *p* = 0.0432)

Concerning responding T-cells, at T0, a lower percentage of responding T-cells in PLWH with <200 cells/µL compared to PLWH with >200 cells/µL was observed (CD4: *p* = 0.0331; CD8: *p* = 0.0055) as well as compared to HD (CD4: *p* = 0.0295; CD8: *p* = 0.0056) (Figure 2D).

At T1, no statistically significant differences were found in the percentage of responding T-cells between the stratified subgroups, as well as between the stratified subgroups and HD (2D).

All medians and [IQR] are reported in Table S3.

### 2.4. Evaluation of Spike-Specific Humoral and T-Cell Response in PLWH Stratified According to Humoral Response

**Figure 3:**
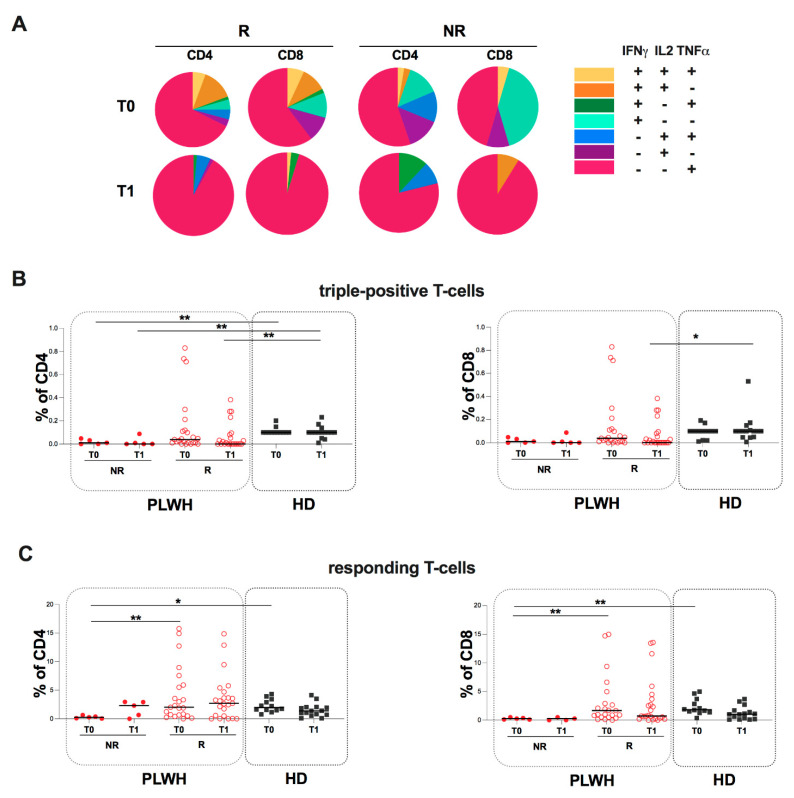
Evaluation of S-specific humoral and T-cell response in PLWH stratified according to humoral response into NR and R at T0 and T1. (**A**) Pie charts representing multifunctional cytokine analysis of specific CD4 and CD8 T-cells in PLWH stratified according to humoral response. (**B**) Percentage of triple-positive T-cells in PLWH stratified according to humoral response. The differences between NR and R subgroups were evaluated using the nonparametric Mann-Whitney test. Both NR and R subgroups were compared to HD using the nonparametric Kruskal-Wallis test with Dunn’s post-test. Data are shown as median (lines). (**C**) Percentage of responding T-cells in PLWH stratified according to humoral response. The differences between NR and R subgroups were evaluated using the nonparametric Mann-Whitney test. Both subgroups were compared to HD using the nonparametric Kruskal-Wallis test with Dunn’s post-test. Data are shown as median (lines). Statistical significance (p) is reported in the graphics. ** *p* < 0.01; * *p* < 0.05.

Regarding T-cell subset distribution, stratifying PLWH according to humoral response into NR (5 male and 0 female, median age [IQR] 62 [44.5–66.5]) and R (21 male and 11 female, median age [IQR] 61 [48–68]), at both time-points, no differences were found in CD4 subset distribution between R and NR subgroups (Figure 3B).

In particular, in both subgroups, the proportion of monofunctional T-cells producing TNFα (IFNγ-IL2-TNFα+) is the most represented with respect to the other subpopulations.

At T0, concerning triple-positive T-cells, no statistically significant differences were found between the stratified subgroups (Figure 3B).

Comparing NR and R subgroups to HD, a lower percentage of triple-positive was found in the NR subgroup compared to HD (CD4: *p* = 0.0093) (Figure 3B). At T1, comparing the two subgroups to HD, a lower percentage was found in the NR subgroup compared to HD (CD4: *p* = 0.0098) as well as in the R group compared to HD (CD4: *p* = 0.0030; CD8: *p* = 0.0473) (Figure 3B).

Concerning responding T-cells, at T0, a lower percentage of responding T-cells in the NR subgroup compared to the R one was observed (CD4: *p* = 0.0052; CD8: *p* = 0.0064) as well as compared to HD (CD4: *p* = 0.00117; CD8: *p* = 0.0063) (Figure 3C). Otherwise, at T1, no statistically significant differences were found in the percentage of responding T-cells between the subgroups (Figure 3C).

All medians and [IQR] are reported in Table S4.

### 2.5. Correlations between Current CD4 Cell Count and T-Cell Response

**Figure 4:**
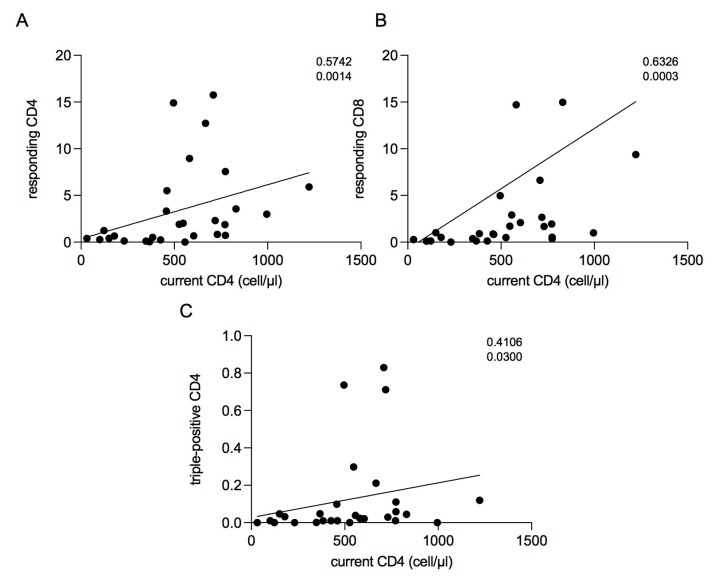
Correlations. (**A**) Positive correlation between CD4 current cell count and responding CD4 T cells (ρ = 0.5742 *p* = 0.0014). Linear correlation was evaluated by using the regression test. (**B**) Positive correlation between CD4 current cell count and responding CD8 T cells (ρ = 0.6326 *p* = 0.0003). Linear correlation was evaluated by using the regression test. (**C**) Positive correlation between CD4 current cell count and triple-positive CD4 T cells (ρ = 0.4106 *p* = 0.0300). Linear correlation was evaluated by using the regression test. Linear correlation was evaluated by using the regression test. All correlations were performed using Spearman’s test.

Overall, we found a weak positive correlation between the current CD4 cell count and responding T-cells (CD4: ρ = 0.5742, *p* = 0.0014; CD8: ρ = 0.6326; *p* = 0.0003), and a positive correlation between current CD4 cell count and triple-positive CD4 T-cells (ρ = 0.4106, *p* = 0.0300) (Figure 4A–C).

### 2.6. Evaluation of Spike-Specific Humoral and T-Cell at T2 in PLWH

**Figure 5:**
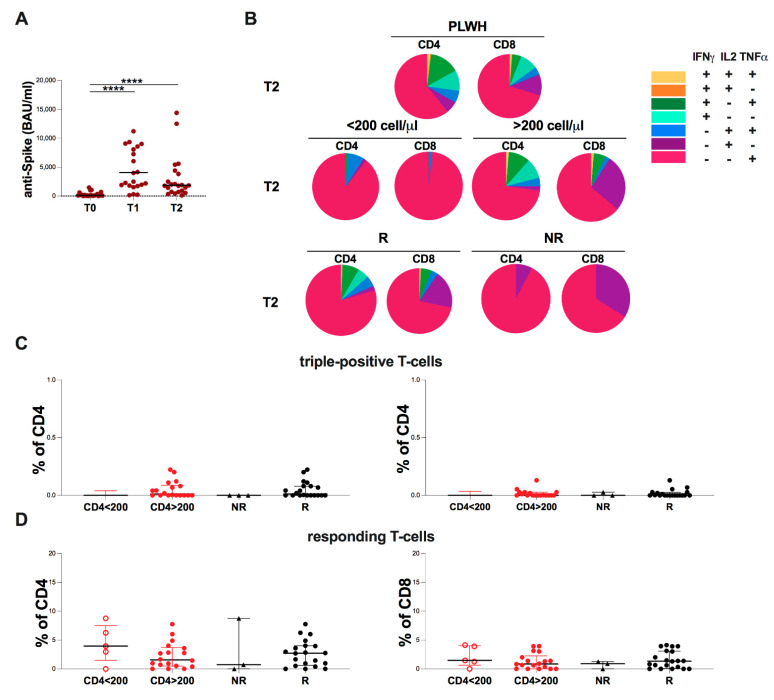
Evaluation of S-specific humoral and T-cell response in PLWH at T2. (**A**) Anti-S antibody levels in PLWH. The cross-sectional evaluation was made using the nonparametric Mann-Whitney test. Data are shown as median (lines). (**B**) Frequency of CD4 and CD8 T cells producing all combinations of IFNγ, IL2, and TNFα in PLWH and in PLWH stratified according to CD4 cell count and according to humoral response into NR and R. (**C**) Percentage of triple-positive T-cells levels in PLWH stratified according to current CD4 cell count and humoral response. The differences between CD4 > 200 and CD4 < 200 and between R and NR groups were evaluated using the nonparametric Mann-Whitney test. Data are shown as median (lines). (**D**) Percentage of responding T-cells levels in PLWH stratified according to current CD4 cell count and humoral response. The differences between CD4 > 200 and CD4 < 200 and between R and NR groups were evaluated using the nonparametric Mann-Whitney test. Data are shown as median (lines). Statistical significance (p) is reported in the graphics. **** *p* < 0.0001.

As reported in Figure 5A, the cross-sectional evaluation of S-specific humoral response in PLWH showed significantly higher levels at T1 and T2 compared to T0 (*p* < 0.0001 and *p* < 0.0001, respectively) (Figure 5A). Analyzing the subset distribution, in PLWH, the proportion of CD4 and CD8 monofunctional T-cells producing TNFα (IFNγ-IL2-TNFα+) was the most represented, and the same result was also maintained after the stratification, with no statistical differences in the subset distribution between the stratified subgroups.

At T2, we found no statistically significant differences in the percentage of triple-positive and responding T-cells between PLWH with <200 cells/µL and >200 cells/µL subgroups as well as between NR and R (Figure 5B,C).

All medians and [IQR] are reported in Table S5.

## 3. Discussion

In this study, we aimed to contribute to clarifying the knowledge gaps in the understanding of the quality, magnitude, and duration of immunity to SARS-CoV-2 vaccination in PLWH, which is critical for the proper application of mitigation strategies, including additional dose strategies, as well as for vaccine design [22]. Therefore, we evaluated humoral and T-cell response to the SARS-CoV-2 vaccine in this population before and after the third dose of the Comirnaty^®^ vaccine, according to current CD4 cell count and humoral response, comparing the obtained findings with a control group (HD).

Our results showed that a specific antibody response was present in most (86%) of PLWH already after the second dose and the levels of anti-S were comparable between them and HD, indicating the ability to mount a specific humoral immune response [13]. However, the third dose increased both the rate of response and its magnitude.

In addition to antibodies, it has previously been proved that T-cells in convalescent individuals provide protective roles in controlling SARS-CoV-2 infection reducing viral replication and limiting the pathogenicity of infection [23]. This has determined increasing attention on vaccine-induced S-specific T-cells, which are capable of recognizing different regions of S protein, contributing to vaccine efficacy against viral variants [8,9].

In detail, we investigated T-cell response by intracellular cytokine flow cytometry assay upon S peptide libraries stimulation, and through the Boolean gating, we characterized all the possible combinations of the intracellular expression of IFNγ, IL2 and TNFα in T-cells, identifying those producing any of them as responding T-cells and those producing all of them simultaneously as triple-positive T-cells.

First of all, we analyzed the T-cell subset distribution, evidencing an uneven T-cell subset distribution in PLWH at both time points, different from the heterogeneous one found in HD. In particular, we observed that the subset distribution of IFNγ, IL2 and TNFα in PLWH was dominated by a higher proportion of monofunctional T-cells producing TNFα (IFNγ-IL2-TNFα+ T-cells) and lower levels of triple-positive T-cells. Since the quality of T-cell response refers to the combination of functions that T-cells are able to exert and that a higher degree of multifunctionality has been associated with higher protection, we speculate that the highly uneven subset distribution of T-cells found in PLWH could indicate a qualitatively inferior T-cell response compared to that of HD [22,24]. On the contrary, as expected, the subset distribution of IFNγ, IL2 and TNFα of CD4 T-cells in HD was heterogeneous, characterized by a higher proportion of IFNγ (CD4 IFNγ+IL2-TNFα-), IL2 (CD4 IFNγ-IL2+TNFα-), and TNFα (CD4 IFNγ-IL2-TNFα+) CD4 producing T-cells, indicating a Th1 polarization of antigen-specific CD4 T-cells, that are known to exert a protective role against viral infections and other intracellular pathogens and supporting the ability of the mRNA vaccine to induce a coordinated immune response in this population [25]. These findings are consistent with the study conducted by Arunachalam et al., which found that vaccination elicited an expansion of IFNγ CD4 T-cells with a preserved multifunctionality [26]. The subset distribution of IFNγ, IL2 and TNFα CD8 T-cells was heterogeneous and in line with the CTL activity explicated by these cells [27]. Furthermore, a T-cell response characterized by a limited expression of IFN-γ, low levels of polyfunctional T-cells, and high levels of monofunctional T-cells producing TNF-α has been associated with COVID-19 severity, suggesting that in PLWH, the coinfection could exacerbate COVID-19 pathology [28,29].

However, analyzing the percentage of responding T-cells, we found no differences between PLWH and HD at both time points, indicating the presence of a response, although attributable predominantly to that of monofunctional T-cells producing TNFα.

Contrary to what is reported in other studies, we didn’t find a relation between humoral response and CD4 cell count [19,21]. In fact, stratifying the population according to this parameter, we didn’t observe any differences in anti-S levels between the stratified subgroups as well as between them and HD.

Analyzing the T-cell subset distribution, we found that the same pattern found in PLWH, characterized by a higher proportion of monofunctional T-cells producing TNFα, was also maintained after the stratification of PLWH according to CD4 cell count also in >200 cell/µL subgroup, indicating a persistent immunological signature despite successful ART, as well as a lower proportion of triple-positive T-cells in both subgroups with respect to HD.

Concerning responding T-cells, we found that after the second dose, in PLWH with >200 cell/µL, the percentage was similar, in terms of magnitude and durability, to those found in HD, while it was significantly lower in PLWH with <200 cell/µL. These data are in line with previous reports by Antinori et al. that suggest that the immunogenicity at the time of vaccination was related to the current CD4 cell count [21].

After the administration of the third dose, the levels of responding T-cells in PLWH with <200 cell/µL were comparable to those of PLWH with >200 cell/µL and HD, highlighting how the third dose improved the responsiveness to vaccination in frail patients with severe immune impairment in terms of magnitude. The same result was found also stratifying the population into R and NR, suggesting the presence of a cell-mediated response in the absence of a humoral one. However, this response is again attributable to that of monofunctional IFNγ-IL2-TNFα+ T-cells.

Overall, we found positive correlations between the current CD4 cell count and activated T-cells as well as with polyfunctional CD4 T-cells, supporting the idea that the immunogenicity at the time of vaccination was related to the current CD4 cell count [21].

The specific humoral response seems to last up to six months from the administration of the third dose [13,30]. Regarding T-cell subset distribution, we found that the presence of a higher proportion of IFNγ-IL2-TNFα+ monofunctional T-cells was maintained in PLWH globally as well as in the stratified subgroups. Furthermore, the levels of triple-positive and responding T-cells remained comparable between the stratified subgroups.

This study has several limitations, including the low number of HD and the various sample sizes in the subgroups after the stratification of PLWH. Moreover, we didn’t evaluate the neutralizing activity of the antibodies nor the efficacy of vaccination against SARS-CoV-2 viral variants.

Collectively, our data support the hypothesis that mRNA vaccination is able to elicit a humoral and cellular immune response against SARS-CoV-2 in most PLWH receiving ART, although the latter seems to be qualitatively different compared to that of HD.

Therefore, a fundamental comprehension of the dynamic of T-cell quality is still needed since it is the quality of the response rather than its magnitude that may represent an important correlate of protection against infections [24,31].

This has implications for the mitigation strategies against the current pandemic and for the design of future vaccines in general; it would be helpful to better understand the capability of immune response in ART-treated PLWH.

## 4. Materials and Methods

### 4.1. Study Design and Participants

PLWH were enrolled in this study from September 2021 to February 2022. The cohort included individuals with HIV who were stable on ART under routine follow-ups at the Infectious Diseases Unit of S.M Goretti Hospital of Latina, Italy, and received Comirnaty^®^ vaccination according to the schedule of attendance in the context of the Italian national vaccination program. HIV viral load and CD4 cell count were routinely assessed. Blood samples were collected using ethylenediaminetetraacetic acid (EDTA) tubes and in tubes with gel for serum separation on the same day (T0) and after two months (T1) from the administration of the third dose. To compare humoral and T-cell response before (T0) and after the booster dose (T1), healthy donors (HD) vaccinated with two doses of Comirnaty^®^ were also included in the study as the control group. For PLWH, an additional time point of six months from the administration of the third dose was considered (T2).

All enrolled PLWH were stratified according to CD4 cell count into two subgroups: CD4 < 200 cells/µL and CD4 > 200 cells/µL, as well as according to humoral response into Non-Responder (NR) and Responder (R) subgroups. The differences in humoral and specific T-cell response were evaluated among the stratified subgroups: PLWH with <200 cells/µL versus >200 cells/µL and NR versus R.

### 4.2. Evaluation of Anti-S Antibodies

Specific SARS-CoV-2 total anti-S IgG antibodies were measured on serum samples with a commercial chemiluminescence immunoassay (CLIA), the DiaSorin Liaison SARS-CoV-2 TrimericS IgG (DiaSorin TriS IgG; DiaSorin S.p.A, Saluggia, Italy). Performance of sensitivity and specificity were reported according to the manufacturer’s instructions. The levels of anti-SARS-CoV-2 IgG antibodies were expressed in the World Health Organization International Standard (NIBSC code. 20/268) binding arbitrary unit (BAU/mL). Samples with values of ≥33.8 BAU/mL were considered positive.

The lower detection limits of the assay were 4.81 BAU/mL.

### 4.3. Determination of Anti-N Antibodies

The presence of anti-N against SARS-CoV-2 was determined on serum samples using the KT-1032 EDI^TM^ Novel Coronavirus COVID-19 IgG Enzyme-Linked Immunosorbent Assay (ELISA) Kit (Epitope Diagnostics, Inc. 7110 Carroll Rd, San Diego, CA 92121, USA) and performed according to the manufacturer’s instructions, to exclude any possible pre-exposure to asymptomatic natural SARS-CoV-2 infection.

According to the manufacturer, the average value of the absorbance of the negative control is less than 0.25, and the absorbance of the positive control is not less than 0.30.

### 4.4. T-Cell Stimulation with SARS-CoV-2–Specific Peptide Libraries

Peripheral blood mononuclear cells (PBMC) were isolated, as previously described [32]. Isolated PBMC was cryopreserved in cell recovery media containing 10% of dimethyl sulfoxide (DMSO), supplemented with heat-inactivated Fetal Calf Serum (FCS), and stored at −196 °C until used in the assay.

As previously described [33,34], T-cell specific response was assessed on isolated PBMC using multiparametric flow cytometry after overnight stimulation with SARS-CoV-2 peptide libraries. Pools of lyophilized peptides, covering the immunodominant sequence domains of the Spike glycoprotein (S) (GenBank MN908947.3, Protein QHD43416.1) and the Nucleocapsid phosphoprotein (N) (GenBank MN908947.3, Protein QHD43423.2) of SARS-CoV-2 were purchased from Milteny Biotec (Milteny Biotec, Germany). Specifically, PepTivator SARS-CoV-2 Prot_S1 covered the N-terminal S1 domain of the spike protein (amino acids [aa] 1–692). PepTivator SARS-CoV-2 Prot_S covered selected immunodominant sequence domains of the spike protein (aa 304–338, 421–475, 492–519, 683–707, 741–770, 785–802, and 885–1273). PepTivator SARS-CoV-2 Prot_N covered the complete sequence of the N phosphoprotein of SARS-CoV-2. For each patient, a negative and positive (phytohemagglutinin [PHA] 5 μg/mL) control was also included.

### 4.5. Flow Cytometry Assay of Stimulated T-Cells

Surface and intracellular staining of stimulated T-cells by flow cytometry were made following a previously published protocol from our group [34]. Briefly, for the surface staining PacificBlue-conjugated anti-CD45 to identify cells belonging to lymphoid and myeloid lines, APC-Cy7-conjugated anti-CD4 and APC-conjugated anti-CD8 and APC-Cy7-conjugated anti-CD4 to identify CD4+ and CD8+ T-cells, respectively, were used.

For intracellular staining, FITC-conjugated anti-IFNγ, PerCp-Cy5.5-conjugated anti-TNFα, and PE-Cy7-conjugated anti-IL2 were used. All antibodies were from BioLegend (BioLegend, San Diego, CA, USA) and used according to the manufacturer’s instructions.

The stained samples were acquired using the MACSQuant Flow Cytometer (Miltenyi Biotec, Germany) and analyzed using FlowJo™ v10.8.1 software, and the cytokine background obtained from the negative condition (unstimulated) was subtracted from the stimulated ones.

Co-expression of cytokines was analyzed via Boolean gating using FlowJo™ v10.8.1. We identified T-cells producing any of IFNγ or IL2 or TNFα as responding T-cells and triple-positive T-cells those simultaneously producing all 3 cytokines (IFNγ+IL2+TNFα+ T cells). Analysis of the different cytokine distributions was performed using SPICE v6.1 software.

### 4.6. Ethics Statement

This study was approved by the Local Ethics Committee (protocol number 0062/2022). Each subject gave written informed consent for participation in the study.

### 4.7. Statistical Analysis

All statistical analyses were performed using GraphPad Prism v.9 software for macOS, and two-tailed *p* ≤ 0.05 was considered statistically significant. Values are reported as the median and interquartile range (IQR). The nonparametric comparative Mann-Whitney test and the nonparametric Kruskal-Wallis test with Dunn’s post-test were used for comparing medians between groups. Longitudinal evaluation of anti-S levels was performed using the nonparametric Wilcoxon test. Spearman’s rank correlation analysis was used to assess the relationship between clinical and laboratory data and CD4 cell count (Spearman’s coefficient [r] and statistical significance [p] are reported in the graphics). Distribution differences of the different cytokine combinations between groups and sub-groups were performed using the nonparametric Permutation test using SPICE, distributed by the National Institute of Allergy and Infectious Diseases, NIH.

## Figures and Tables

**Table 1:** Demographic and clinical characteristics of the study population.

	PLWH (n = 37)	HD (n = 18)
Age, median (IQR) years	61 (48–68)	30 (30–53)
Male/Female	26/11	13/5
Current CD4 cell count (cells/µL)	547 (308–714)	
CD4+ cell count nadir, (cells/µL)	90 (22–281)	
HIV-RNA zenith (cp/mL)	92,280 (17,000–544,323)	
HIV-RNA (cp/mL)	40 (40–166)	
ART		
Dual (%)	8/37 (22%)	
Non-Dual (%)	29/37 (78%)	
Comorbidities		
Any (%)	11/37 (30%)	
More than one (%)	26/37 (70%)	
HCV serostatus		
Anti-HCV Ab positive (%)	9/37 (24%)	
Smoke status		
No (%)	24/37 (65%)	
Yes (%)	13/37 (35%)	

Data are showed as median (IQR) or no. (%) of subjects. n: number; IQR: interquartile range; ART: antiretroviral therapy; HCV: Hepatitis C Virus; Ab: antibody.

## Data Availability

The raw data supporting the conclusions of this article will be made available by the authors without undue reservation.

## Supplementary Materials

The following supporting information can be downloaded at: https://www.mdpi.com/article/10.3390/ijms232314988/s1.
