# Supplementary material for: Quality of T-Cell Response to SARS-CoV-2 mRNA Vaccine in ART-Treated PLWH
Source: Int J Mol Sci. 2022 Nov 30;23(23):14988. doi: 10.3390/ijms232314988 (PMC9741180; doi:10.3390/ijms232314988)
Supplement: Supplementary file 1 [file ijms-23-14988-s001.zip › ijms-2043352-supplementary.pdf]

Table S1. Demographic and clinical characteristics of study population at each time-point

	PLWH (n=37)			HD (n=18)	
	T0	T1	T2	T0	T1
Number	31	28	27	12	15
Age, median (IQR) years	62 (48-68)	63 (56-69)	61 (47-68)	42 (33-53)	38 (30-52)
Male/Female	23/8	21/7	18/9	4/8	3/12
Current CD4 cell count (cells/μl)	557 (367-719)	536 (353-727)	495 (231-719)		
CD4+ cell count nadir, (cells/μl)	119 (22-310)	146 (24-305)	80 (13-310)		
HIV-RNA zenith (cp/ml)	86100 (16957-508750)	75010 (15260-508750)	69251 (15260-264883)		
HIV-RNA (cp/ml)	82 (40-167)	40 (40-164)	124 (40-510)		
ART					
Dual (%)	8/31 (26%)	7/28 (25%)	8/27 (30%)		
Non-Dual (%)	25/31 (74%)	21/28 (75%)	19/27 (70%)		
Comorbidities					
Any (%)	7/31 (23%)	6/28 (21%)	4/27 (15%)		
More than one (%)	24/31 (77%)	22/28 (79%)	23/27 (85%)		
HCV serostatus					
anti-HCV Ab positive (%)	7/31 (23%)	7/28 (25%)	5/27 (19%)		
Smoke status					
No (%)	22/31 (71%)	20/28 (71%)	22/27 (81%)		
Yes (%)	9/31 (29%)	8/28 (29%)	5/27 (19%)		

Table S2. Medians and IQR of immunological parameters in PLWH and HD at T0 and T1

	PLWH		HD	
	T0	T1	T0	T1
Anti-S antibody levels	156 [66-330.50]	2770 [1730-8570]	369 [189-700.50]	1660 [1520-9400]
% of triple-positive CD4	0.03 [0.01-0.11]	0.0 [0.0-0.07]	0.10 [0.10-0.10]	0.10 [0.10-0.10]
% of triple-positive CD8	0.02 [0.0-0.12]	0.01 [0.0-0.10]	0.10 [0.04-0.10]	0.10 [0.05-0.11]
% of responding CD4	1.56 [0.39-5.01]	2.67 [0.53-3.65]	1.98 [1.52-3.29]	1.39 [0.70-2.05]
% of responding CD8	0.95 [0.36-2.85]	0.68 [0.19-3.43]	1.82 [1.42-3.48]	1.02 [0.17-1.70]

Table S3. Medians and IQR of immunological parameters in PLWH stratified according to CD4 cell count

	<200 cells/μl		>200 cells/μl	
	T0	T1	T0	T1
Anti-S antibody levels	153 [4.81-613]	3788 [102.50-12953]	156 [78.25-322]	2770 [1850-8795]
% of triple-positive CD4	0.01 [0.0-0.04]	0.0 [0.0-0.16]	0.04 [0.01-0.14]	0.0 [0.0-0.06]
% of triple-positive CD8	0.0 [0.0-0.04]	0.0 [0.0-0.20]	0.04 [0.0-0.15]	0.0 [0.0-0.12]
% of responding CD4	0.40 [0.24-0.81]	2.38 [0.0-3.03]	2.17 [0.64-6.32]	2.82 [0.57-4.88]
% of responding CD8	0.20 [0.08-0.64]	0.57 [0.21-1.16]	1.69 [0.52-5.39]	0.77 [0.18-4.84]

Table S4. Medians and IQR of immunological parameters in PLWH stratified according to humoral response

	NR		R	
	T0	T1	T0	T1
% of triple-positive CD4	0.01 [0.0-0.04]	0.0 [0.0-0.05]	0.04 [0.01-0.12]	0.0 [0.0-0.08]
% of triple-positive CD8	0.01 [0.0-0.05]	0.0 [0.0-1.70]	0.04 [0.0-0.14]	0.0 [0.0-0.11]
% of responding CD4	0.28 [0.07-0.53]	2.3 [0.34-2.94]	2.02 [0.68-5.91]	2.7 [0.51-4.70]
% of responding CD8	0.27 [0.11-0.44]	0.27 [0.0-17.26]	1.68 [0.53-4.98]	0.72 [0.24-3.67]

Table S5. Medians and IQR of immunological parameters in PLWH and in the stratified subgroups at T2

	PLWH				
	TOT	<200 cells/μl	>200 cells/μl	NR	R
Anti-S antibody levels	1850 [724-5430]	12500 [840-31450]	1770[715-3160]	0.0 [0.0-0.0]]	0.01 [0.0-0.08]
% of triple-positive CD4	0.0 [0.0-0.08]	0.0 [0.0-0.03]	0.01 [0.0-0.09]	0.0 [0.0-0.03]	0.0 [0.0-0.08]
% of triple-positive CD8	0.0 [0.0-0.03]	0.0 [0.0-0.03]	0.0 [0.0-0.03]	0.73 [0.0-8.80]	2.73 [0.63-4.01]
% of responding CD4	2.70 [0.0-4.0]	0.0 [0.0-0.03]	1.55 [0.49-3.70]	0.90 [0.0-1.30]	1.35 [0.08-3.09]
% of responding CD8	1.30[0.0-3.02]	1.47 [0.64-4.02]	0.86 [0.0-2.26]	0.0 [0.0-0.0]]	0.01 [0.0-0.08]
